# Reactive Oxygen, Nitrogen, and Sulfur Species (RONSS) as a Metabolic Cluster for Signaling and Biostimulation of Plants: An Overview

**DOI:** 10.3390/plants11233203

**Published:** 2022-11-23

**Authors:** Julia Medrano-Macías, Adriana Carolina Flores-Gallegos, Erika Nava-Reyna, Isidro Morales, Gonzalo Tortella, Susana Solís-Gaona, Adalberto Benavides-Mendoza

**Affiliations:** 1 Department of Horticulture, Universidad Autónoma Agraria Antonio Narro, Saltillo 25315, Mexico; 2 Bioprocesses and Bioproducts Research Group, Food Research Department, School of Chemistry, Universidad Autónoma de Coahuila, Saltillo 25280, Mexico; 3 Instituto Nacional de Investigaciones Forestales, Agrícolas y Pecuarias, National Center for Disciplinary Research in Water, Soil, Plants and Atmosphere Relations, Gomez Palacio 35150, Mexico; 4 Instituto Politécnico Nacional, Interdisciplinary Research Center for Regional Integral Development, Oaxaca 71230, Mexico; 5 Centro de Excelencia en Investigación Biotecnológica Aplicada al Medio Ambiente (CIBAMA-BIOREN), Facultad de Ingeniería y Ciencias, Universidad de La Frontera, Temuco 4811230, Chile; 6 UPL, Saltillo 25290, Mexico

**Keywords:** biostimulants, redox homeostasis, plant stress, tolerance inductors, elemental sulfur, sulfur nanoparticles, nitric oxide, ROS, RNS, RSS

## Abstract

This review highlights the relationship between the metabolism of reactive oxygen species (ROS), reactive nitrogen species (RNS), and H_2_S-reactive sulfur species (RSS). These three metabolic pathways, collectively termed reactive oxygen, nitrogen, and sulfur species (RONSS), constitute a conglomerate of reactions that function as an energy dissipation mechanism, in addition to allowing environmental signals to be transduced into cellular information. This information, in the form of proteins with posttranslational modifications or signaling metabolites derived from RONSS, serves as an inducer of many processes for redoxtasis and metabolic adjustment to the changing environmental conditions to which plants are subjected. Although it is thought that the role of reactive chemical species was originally energy dissipation, during evolution they seem to form a cluster of RONSS that, in addition to dissipating excess excitation potential or reducing potential, also fulfils essential signaling functions that play a vital role in the stress acclimation of plants. Signaling occurs by synthesizing many biomolecules that modify the activity of transcription factors and through modifications in thiol groups of enzymes. The result is a series of adjustments in plants’ gene expression, biochemistry, and physiology. Therefore, we present an overview of the synthesis and functions of the RONSS, considering the importance and implications in agronomic management, particularly on the biostimulation of crops.

## 1. RONSS Integration as a Metabolic Cluster

Plant metabolism consists of a conglomerate of chemical reactions in which free energy is dissipated from physical sources such as radiation or chemical sources that store energy in chemical bonds or chemical potentials. What is obtained in organisms is metabolic energy, biomolecules, and information to maintain cellular, tissue, and organ structures in a dynamic steady state.

Cellular metabolism processes are believed to be descendants of ancient abiotic processes that dissipate free energy from physical and chemical sources, which occurred before the emergence of organized cell life [1,2,3]. Such abiotic processes are supposed to have arisen spontaneously as one of several physicochemical mechanisms through which the primordial Earth system dissipated free energy from the Sun or the stores of substances in the Earth’s crust [4,5]. It is thought that many of these processes occurred through reactions that involved the transfer of electrons [6], which could partly explain the preponderance of redox processes in the metabolism of modern organisms [7].

The goal of the above processes was to maximize entropy generation from free energy [4,8]. One way to maximize the entropy produced is to carry out cooperative work between different molecular species, which implies the collective organization of diverse functions in conglomerates or clusters [1,4]. It can be assumed that molecular conglomerates functioned as collaborative energy-channeling mechanisms. Different molecular complexes probably organized themselves to transfer energy from one molecular species to another, making the process of energy dissipation (or entropy generation) more effective than the result of the individual functions [9]. The chemical conglomerates or clusters were dedicated to dissipating free energy in the form of reduction potential to produce reactive chemical species such as reactive oxygen species (ROS), reactive nitrogen species (RNS), and reactive sulfur species (RSS), collectively termed reactive oxygen, nitrogen, and sulfur species (RONSS).

**Figure 1:**
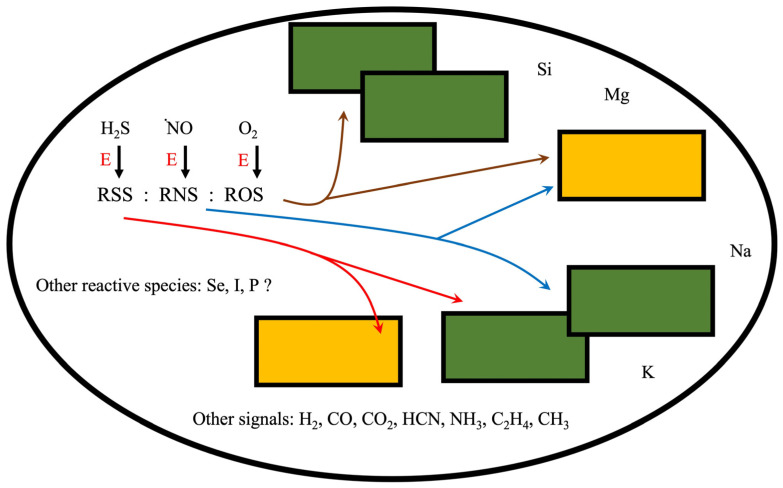
Schematic representation of a prebiotic supramolecular complex that processes energy and matter [1]. The different components represented by the colored rectangles carried out specialized functions and interacted with each other coordinating through energy signals (redoxtasis) and chemical signals created by metabolites. It is possible that the RSS:RNS ratio, and later the RSS:RNS:ROS ratio, modified the redox homeostasis of the prebiotic system, modified internal signals, and caused changes in the nucleic acids, proteins, peptides, and other organic molecules of the prebiotic supramolecular complexes [12,13]. E: energy; RSS: reactive sulfur species; RNS: reactive nitrogen species; ROS: reactive oxygen species.

The cooperative work of the different components in different compartments required the creation of networks for the transmission of endogenous information that improved the ability to adjust to the conditions of the environment [1,4,10]. These information networks, which could include organic and inorganic soluble and volatile compounds (Figure 1), were possibly the ancestors of cell signaling processes. In Figure 1, RONSS resulting from the energy dissipation by H_2_S, **·**NO, and inorganic and organic compounds (and lately O_2_) became part of the information system that monitored the energy state or redoxtasis of the different processes, regulating the joint action of the different components. In particular, the RSSH derived from the interaction of H_2_S with thiols could be, due to their amphiphilic nature, chemical agents that increased the system’s flexibility in terms of the degrees of freedom available for the flow of electrons. Some elements such as K, Mg, Fe, Na, and Si possibly formed activation or protection systems for various components of the system. It is possible that some abundant elements, such as Fe and other heavy metals, have not been used in more significant volumes due to their ability to trigger oxidative reactions, which could cause system instability due to RONSS saturation. Therefore, Fe in biological organisms functions as a trace element.

Compartmentalization gave rise to more sophisticated systems for copying structures, the precursors of reproduction systems, probably based on the ability to store information on functional patterns through the emergence of Hopfield-like attractor dynamics [11]. Such compartmentalization may have given rise to the first cellular organisms with different metabolic abilities, according to the energy and matter use niche in which they evolved [1].

As a consequence of the above, the metabolic processes of living organisms not only function as a mechanism to maintain the structure and functions of organisms but, as a consequence of their intrinsic dissipative nature, they still operate cooperatively to maximize the generation of entropy [8,14]. Metabolism is the set of biochemical processes that, in addition to processing matter and information, allows the acquisition, transformation, and dissipation of free energy available in the environment. Metabolism comprises a set of supramolecular conglomerates or clusters that work cooperatively, giving rise to the different phenomena that allow cellular life. The metabolic pathways that produce reactive species of certain elements, such as S (RSS), N (RNS), and O (ROS), can be an example of the above since they are linked to energy metabolism, functioning as dissipative processes of the reduction potential in excess [15,16] and can, to a certain extent, be visualized as a cluster of processes with diverse functions: the primary being energy dissipation, followed by information transfer or signaling. The dissipative processes possibly did not initially have a goal of regulation or control of the redoxtasis but were spontaneous processes for energy dissipation. Their use as regulatory or signaling agents may be a later adaptation [17].

Other inorganic reactive species, e.g., I, Se, and P reactive species, and RONSS-derived reactive species such as lipid hydroperoxides (LOOH), carbonyl species (RCS), and malondialdehyde (MDA), have similar signaling functions [18,19,20,21,22,23]. However, they may operate at smaller concentrations than S, N, and O reactive species.

Perhaps initially with a preponderance of the RSS (H_2_S) and RNS (**·**NO) during the long Archean anoxygenic phase of planetary evolution, to later incorporate ROS [24,25], when O_2_ increased its concentration during the Proterozoic phase of Earth’s evolution [6,26,27]. However, if O_2_ or oxygen compounds such as H_2_O_2_ were present as traces before the complete oxygenic phase ([atmospheric O_2_] > 2%) [28], they could be sources of ROS. In the latter case, the joint evolution of the RONSS could have started before the concentration of O_2_ rose substantially.

The final integration and cooperation of RONSS may result, through the self-organization and creation of novelties that characterize complex systems [29], in the obtention of cooperative systems to transform free energy into information [30,31]. The information accumulated in the dynamic structures and the complexes of structures coordinated through signaling allowed the synchronization of the activities of the metabolism: first, coordinated abiotic processes, and later cellular metabolism [6,7,32,33].

**Figure 2:**
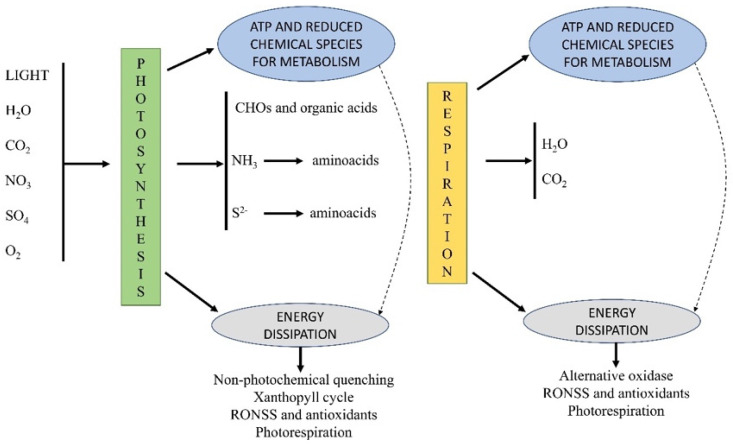
Model of different energy capture and dissipation processes. Both photosynthesis and respiration, as well as the metabolism coupled with these activities, constitute dissipative mechanisms. Photosynthesis and respiration are further associated with other photochemical and biochemical energy dissipation pathways, including the production of RONSS. During the abiotic evolutionary process and later during the early biotic evolution, the production of RONSS went from being only a mechanism for the dissipation of free energy, with the consequent generation of entropy, also constituting a mechanism for regulation and transfer of information on redox and energy status between the different components of the system.

Considering the abovementioned assumptions and that the different metabolic pathways for the energy dissipation and matter transformation may have formed cooperative clusters during the prebiotic era, it is to be expected that RONSS constitutes in modern organisms a system tightly coupled and coordinated with the rest of the cellular processes (Figure 2) [7,27,34]. The impact and biological functions of reactive species on plants have been extensively described in the scientific literature for ROS, RNS, and RSS individually [12,22,33,35,36,37,38,39,40,41,42,43,44]. It has been determined to a much lesser extent for the ROS–RNS, ROS–RSS, and RNS–RSS pairs [45,46,47,48,49,50,51,52,53,54,55,56] and to a lesser extent for the RONSS cluster [13,34,57,58,59,59,60,61].

The issue of the agricultural application of the RONSS constitutes, in addition to a fertile field for scientific research, a potential seedbed for the development of innovations in the field of biostimulants for crop production [34,57,60,61]. This manuscript aims to present a brief view of the metabolism of RONSS and their use as plant biostimulants. Different literature sources are presented, which comprise the application of at least two of the various reactive species in priming, signaling, and adaptive processes.

## 2. RONSS in Plant Metabolism

The sources of RONSS for plants are O_2_**·**^−^ from atmospheric O_2_; **·**NO generated mainly from nitrogenous compounds (NO_3_^−^, NO_2_^−^, and amino acids) that the plant takes as nutrients and to a much lesser extent from traces of **·**NO present in the atmosphere; and the H_2_S produced as part of the assimilation of the sulfur compounds that the plant takes as nutrients and to a much lesser extent the traces of H_2_S and other compounds such as dimethyl sulfide (DMS) and carbonyl sulfide (COS) present in the atmosphere.

**Figure 3:**
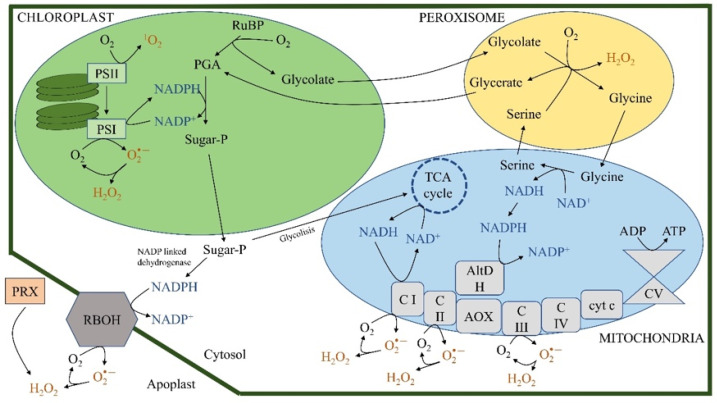
Most important sources of ROS in plant cells. Singlet oxygen (^1^O_2_) may originate from excited triplet chlorophylls (Chl) that activate ground-state O_2_ in the photosystem II (PSII) reaction center. In PSI, superoxide and hydrogen peroxide can be produced by reducing O_2_. In the mitochondrial electron transport chain, complexes CI, CII, and CIII are ROS-generating systems. AltDH, alternative dehydrogenase; AOX, alternative oxidase; cyt. c, cytochrome c; CI-V, mitochondrial complex I–V; PGA, phosphoglycerate; PS, photosystem; PRX, peroxidase; RBOH, respiratory burst oxidase homologs; RuBP, ribulose 1,5-bisphosphate; Sugar-P, sugar–phosphate; TCA, tricarboxylic acid. Modified from [62].

**Figure 4:**
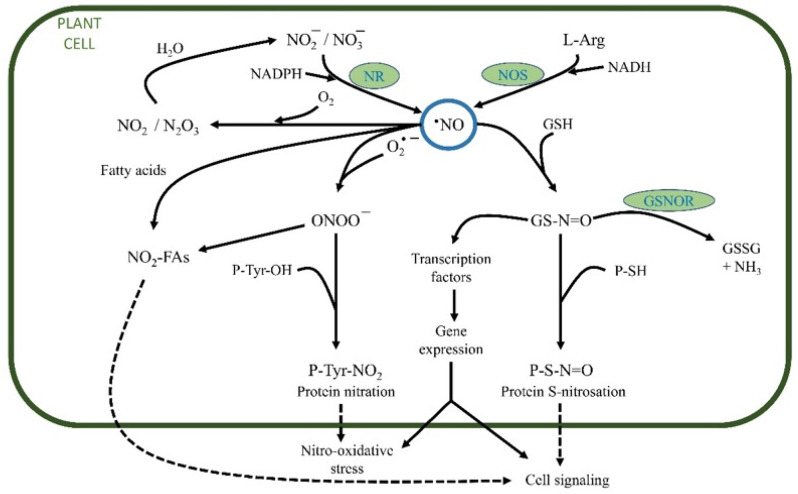
Metabolism of **·**NO in plant cells. **·**NO can be produced by nitrate reductase (NR), L-Arg NO synthase (NOS), or other reductive processes. **·**NO can react by S-nitrosation with glutathione (GSH) to form S-nitrosoglutathione (GS-N=O). GS-N=O can be converted by S-nitrosoglutathione reductase (GSNOR) into oxidized glutathione (GSSG) and NH_3_. As part of the signaling process, the protein sulfhydryl groups can react with GS-N=O and other S-nitrosothiols to produce S-nitrosated proteins (P-S-N=O). Peroxynitrite (ONOO^−^) is an oxidant obtained by interacting **·**NO with O_2_·^−^. The NOOO^−^ can mediate the nitration of proteins (P-Tyr-NO_2_) and fatty acids (NO_2_-FAs). **·**NO in the presence of O_2_ is transformed into N_2_O_3_ and NO_2_, which are subsequently transformed into NO_2_^−^ and NO_3_^−^ in aqueous media. Modified from [40].

**Figure 5:**
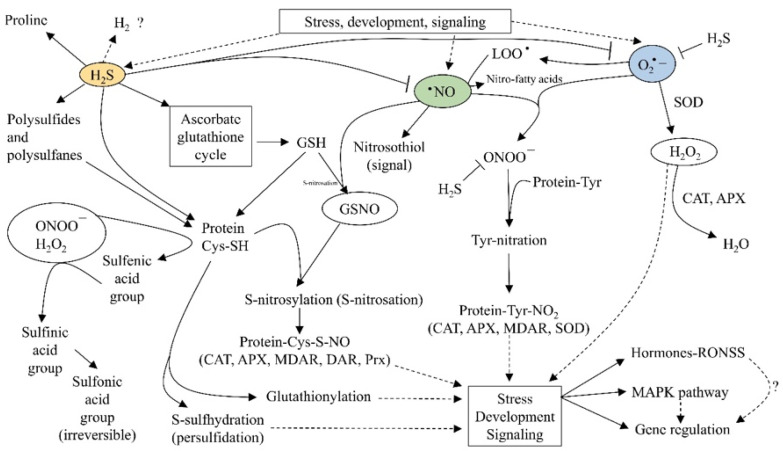
A simplified model of the interactive action of ROS, RNS, and RSS (RONSS) on plant responses during development events or stress-inducing environmental challenges.

Figure 3, Figure 4 and Figure 5 present the transformations in plant cells to obtain the different reactive species, ROS, RNS, and RSS.

### 2.1. Reactive Oxygen Species

Figure 3 illustrates the processes associated with ROS synthesis in plant cells. ROS are the result of a sequential series of one-electron reductions of dioxygen:

O_2_ ← e^−^ → O_2_·^−^ ← e^−^ → O_2_^2−^ ← e^−^ → O_2_^3−^ → O^−^ + e^−^ → O^2−^ (1)

↓ + 2H^+^ ↓ + 2H^+^

H_2_O H_2_O

With the contribution of H^+^, the ROS are transformed as follows:

O_2_^−^ + H^+^ → HO_2_· (perhydroxyl radical) (2)

O_2_^2−^ + 2H^+^ → H_2_O_2_ (3)

O^−^ + H^+^ → OH· (hydroxyl radical) (4)

O^2−^ + 2H^+^ → H_2_O (5)

The above processes allow the dissipation of the excess reducing potential that occurs; for example, in chloroplasts under conditions of high irradiance, or low or high temperature, in mitochondria when low temperatures occur, and in general under any situation that causes an imbalance between the production and the metabolic use of the reducing potential.

The presence of unpaired electrons in O_2_, the high electronegativity (only less than that of F), and the various oxidation states of oxygen (Table 1) explain its ability to accept electrons successively, forming different ROS. S and N are also highly electronegative elements (with S > N), partly explaining their ability to form reactive species.

In addition to their energy dissipation role, ROS act as signaling agents in practically all metabolic and plant development processes [6,63,64]. Interaction with ROS produces peroxidative changes in membranes, protein cysteine and transcription factors, nucleic acids and histones, and low molecular weight metabolites. The above changes modify the functionality of biomolecules, allowing cellular behavior adjustments in response to changes in the redox balance. An example is ROS peroxidation of protein cysteines to sulfenic acid (RSOH). This is a class of oxidative posttranslational modification (oxPTM) of proteins that modifies the redox properties and the capacity for interaction in the cellular environment of the modified protein [6,65]:

RSH + H_2_O_2_ → RSOH + H_2_O (6)

This kind of modification can, for example, change the ability of transcription factors or histones to interact with DNA or the stability or capacity of an enzyme to bind to its substrate. The ROS oxPTM of proteins occurs, for example, in Calvin cycle enzymes, sulfur and starch metabolism, and the proteins hormone-responsive associated with adaptation to stressful environments [66].

The above oxidation of thiols can be reversed using the reducing potential of NADPH:

RSOH + NADPH → RSH + H_2_O + NADP^+^ (7)

acting effectively as a redox switch to move from one protein signaling state to a different one. Most likely, during the prebiotic stage of evolution, this reversible mode of chemical reaction was greatly favored by the ability to dissipate large amounts of free energy using a relatively modest investment in molecular infrastructure.

The epigenomic, proteomic, and metabolomic modifications mentioned above change plants´ phenotype and developmental events, leading to substantial changes in chemical composition, morphology, life cycle, and in general adaptive capacity in a dynamic environment [34,67]. On the other hand, the products of the oxidation of the fatty acids of the membranes, of the thiol groups of the proteins, and several metabolites also constitute a signaling mechanism (through the sensing of the reduction:oxidation balances, e.g., NADPH:NADP^+^, ascorbate:dehydroascorbate, and GSH:GSSH), functioning as a system for perceiving the internal energy states of the system [67,68].

### 2.2. Reactive Nitrogen Species

Nitric oxide (**·**NO) can be considered the primary RNS (Table 2). It is an ancient signaling molecule present in prokaryotes and eukaryotes, including animals and plants [69]. According to Astier et al. [70], although **·**NO is a common chemical theme in the signaling of all living organisms, the way of using the signal to obtain cellular responses (the **·**NO signaling enzymes) seems to have diverged among the different lineages of eukaryotes, and it is different between plants and animals. Those mentioned above may be part of the explanation for the different responses of animals and plants when exposed to RNS. For example, **·**NO_2_ is toxic and is an allergenic agent for animals, but in plants, it is used as a signaling agent [71]. S-nitrosothiols occur as signaling agents in both animals and plants [70]. On the other hand, peroxynitrites are characterized as signaling agents capable of inducing more significant toxicity in animals than in plants. In fact, in mammals can cause guanine nitration leading to mutations and cancer due to guanine mispairs. Meanwhile, in plants, peroxynitrites can be inactivated in the presence of CO_2_, producing CO_3_^−^ and **·**NO_2_. The toxicity of **·**NO is mainly due to the formation of NO-derived oxidants characterized by greater reactivity than **·**NO [72].

In animals, **·**NO is primarily synthesized by nitric oxide synthase [73], while in plants, **·**NO is endogenously produced by different enzyme systems. Among them are the oxidative pathway of the L-Arg NO synthase analogs, by reductive mechanisms such as nitrate reductase (NR) that produces NO_2_ that is reduced to **·**NO by the NR itself, or by the plasma membrane-bound NO-forming nitrite reductase (NOFNiR). The mitochondrial complexes, mainly III and IV, as well as complexes I, II, alternative dehydrogenase, and cytochrome c, also generate **·**NO reductively from NO_2_^−^ [74]. Alternative oxidase (AOX) also produces **·**NO under anoxic or hypoxic conditions in the mitochondria [75], although under normoxia, AOX removes excess **·**NO. Under anoxic conditions and in N_2_ fixation nodules, nonsymbiotic hemoglobins collaborate with mitochondria creating a Phytogb1-NO cycle of **·**NO → NO_3_^−^ → **·**NO that generates anoxic ATP and allows the control of NADPH levels. In addition to NR and NOFNiR, some molybdoenzymes, such as xanthine oxidases, aldehyde oxidases, and sulfite oxidases, seem to possess NO_2_^−^ reductive capacity [22,74,76].

As in the case of ROS, the presence of RNS is associated with dissipation processes of free energy/reducing potential. The preceding is because the main RNS depend on their synthesis on the interaction of **·**NO with the other reactive species that dissipate free energy, ROS, and RSS; secondly, the synthesis of **·**NO is privileged when a plentiful supply of reducing potential (electron pressure) occurs. Electron pressure is substantial, for example, under high irradiance or stress conditions that disturb the flow of electrons in transport chains such as low temperature, water deficit, or salinity. The nonenzymatic reduction of NO_2_^−^ to **·**NO in the presence of high nitrate concentrations in a highly reducing condition or low pH can indeed occur [71,76].

**·**NO generates other SNRs such as peroxynitrite (ONOO^−^), a reaction product between O_2_^−^ and **·**NO [77]. This reaction allows the dissipation of the stored reducing potential resulting from the reduction of NO_3_^−^ to **·**NO:

O_2_^−^ + **·**NO → ONOO^−^ (8)

ONOO^−^ + H^+^ → ONOOH → HO· + **·**NO_2_ → NO_3_^−^ + H^+^ (9)

S-Nitrosothiols are another class of RNS resulting from the reaction of **·**NO with thiol groups, as occurs, for example, when reacting with specific protein sulfhydryl groups to mediate signaling by the S-nitrosated proteins or with H_2_S or glutathione (GSH), to form S-nitrosoglutathione (GS-N=O) [77].

**·**NO + H_2_S → HS-N=O + H^+^ (10)

**·**NO + GSH → GS-N=O + H^+^ (11)

These latter reactions also allow the reduction potential to dissipate. As previously stated, the recovery of the reduced state of thiols requires the consumption of reducing potential (NADH, NADPH, GSH) and the action of the enzyme S-nitrosoglutathione reductase (GSNOR), which catalyzes the irreversible GS-N=O conversion to oxidized glutathione (GSSH) [22].

In addition to their energy dissipation role, RNS are signaling molecules in practically all metabolic and plant development processes (Figure 4). The main mechanisms by which RNS modify cell behavior are through S-nitrosation, nitration, and metal nitrosylation [20,40,77].

S-Nitrosation consists of the formation of S-N=O due to the covalent attachment between **·**NO and the thiol (–SH) of cysteine (Cys). This reversible posttranslational modification (PTM) of proteins is one of the most important mechanisms for NO signaling. The S-N=O group additionally functions as a donor and reservoir of **·**NO. Proteins modified by S-nitrosation change their functionality, inducing rapid and reversible cellular proteome changes [40].

Nitration is the addition, mediated by ONOO^−^, of a nitro group (–NO_2_) into proteins, fatty acids, or nucleic acids. In proteins, the most-studied nitration type results in a nitro-tyrosine formation. However, it also occurs in other amino acids such as cysteine, tryptophan, and methionine. Nitration of amino acids can lead to gain or loss of protein function or even absence of an effect. The most common result is the loss of function [40].

Metal nitrosylation occurs when **·**NO interacts with the transition metals present in proteins. Little information is available on the plants in this process [40].

Similar to ROS, RNS (**·**NO, ONOO^−^, **·**NO_2_) react with fatty acids or LOO**·** (lipid peroxy radicals), forming reactive lipid species called nitro-fatty acids (NO_2_-FAs). NO_2_-FAs constitute signaling molecules and modulate gene expression during stress events and developmental processes [22,78].

### 2.3. Reactive Sulfur Species

The synthesis of H_2_S and other RSS is coupled with the metabolism of S that allows for obtaining the S^2−^ and S^−^ necessary for cellular functions. At the same time, it is a dissipative process that consumes reducing potential, transforming oxidized sulfur species such as S^0^, SO_4_^2−^, SO_3_^2−^, and S_2_O_3_^2−^ into species with a very high reducing potential, such as H_2_S with −0.23 V and glutathione (GSSG/GSH) with −0.24 V [79,80]. The reverse oxidative process, from H_2_S to S^0^ through sequential one-electron oxidations, is the source of RSS (Table 3) such as thiyl radical (HS·), hydrogen persulfide (H_2_S_2_), persulfide ‘supersulfide’ radical (HS_2_·^−^), and elemental sulfur (S^0^) [6].

S0 ← e^−^ → HS_2_^−^ ← e^−^ → H_2_S_2_ ← e^−^→ HS· ← e^−^ → H_2_S (12)

The reducing potential of H_2_S can also be used to reduce disulfides, such as glutathione disulfide (GSSG) and certain protein-based disulfides (PrSSG, PrSSPr). The persulfuration and polysulfuration of protein thiols to obtain persulfides R-S-SH are of great importance in cell signaling [20,81], as well as S^2−^ found in biomolecules and H_2_S, which can be partially oxidized to obtain polysulfides (H_2_S_x_ y S_2_^2−^, S_3_^2−^, S_5_^2−^) that are RSS involved in cell signaling. It appears that H_2_S exerts signaling actions indirectly via H_2_S-derived polysulfides, such as the persulfides RS-SH obtained by the action of H_2_S on thiols and cysteine residues (R-SH), and higher-order polysulfur compounds, i.e., RS_x_H, RS_x_R, with R = glutathione or protein and x ≥ 3. This mechanism can be considered a reversible switch with value to dissipate reducing potential, to signal the redox state of the system, to protect protein thiols from oxidation by ROS (e.g., carbonylation) and to regulate the function of proteins in different metabolic pathways [82]:

RSH (thiol) ← e^−^ → R-S-SH ← e^−^ → R-S_x_-SH (13)

RS-SH contains bound (or sulfane) sulfur, the reactive form of sulfur with a formal oxidation number of −1, but with the capacity of -S-S- to adopt different oxidation states (0 to −2), allowing greater diversity and flexibility of posttranslational modification states in proteins [80,83].

The interaction between thiols and ROS was mentioned previously. The interaction between H_2_S and RNS, e.g., **·**NO, also generates several classes of H_2_S_x_, which seems to establish a direct chemical link between the two reactive molecules [20]. Similarly, GS-N=O when reacting with H_2_S produces **·**NO and a series of RNSS, e.g., SSNO^−^, HSNO, and HNO [84]. Polysulfides can also be obtained by a reductive route using GSH and other RSS (sulfenic acid and thiosulfinates R-S(O)-S-R) and organic polysulfanes (RSS_n_SR, *n* > 2) as precursors [20]. Thiosulfinates are highly reactive toward the thiol groups of GSH and proteins; they are disulfide-S-monoxides found naturally in *Allium* spp. and *Petiveria* spp. Among the thiosulfinates, allicin is one of the most-studied compounds used as a biostimulant, microbicide, and medicine [85]. Organic polysulfanes and those contained in elemental sulfur (S8) and sulfur nanoparticles constitute another group of thiol-reactive compounds with great potential for agricultural use as biostimulants and microbicides [79,86,87,88]. On the other hand, organic polysulfanes (diallyl and dipropyl polysulfanes) subject to reduction can generate RSS- (reduced organic persulfides), which when reacting with GS-N=O produce RSS and **·**NO [89].

Similar to ROS and RNS, RSS are important in cell signaling. Indeed, Olson (2020) [6] notes that RSS has much greater importance than it has been given. The author mentions that RSS includes a more significant amount of reactive chemical species, in addition to the fact that once sulfur is oxidized from its −2 state (H_2_S and S^2−^) to −1, it can be utilized again to reductively regenerate H_2_S from a diversity of organic and inorganic persulfides (RSSH) and polysulfides (H_2_S_x_), e.g., H_2_S_3_, H_2_S_4_, CysSSH, CysSSSH, GSH(Sx)H, GSH(Sx)GSH. Therefore, it is highly likely that a significant source and sink for RSS, compared with ROS and RNS, exists in the cells. Additionally, RSS signaling flexibility is increased by modifying the number of S atoms in persulfides and polysulfides. The higher the number of S atoms, the greater promoted the anionic forms (RSS-) with a nucleophilic character in the terminal S and electrophilic in the nonterminal S, contrary to what occurs with the protonated forms (RSSH) with an electrophilic character in both S atoms [82].

Redox signaling in proteins occurs mainly through redox-sensitive cysteine residues. The -SH group of cysteine has multiple oxidation states (from −2 to +6) that allow a great diversity of modifications when reacting with ROS, RNS, and RSS (Figure 5). The mechanism by which RSS works is called persulfidation:

RSH + H_2_S_2_ → RSSH + H_2_S (14)

Persulfidation is an oxidation that can be reversed through thiol exchange:

R1SSH + R2SH → R1SH + R2SSH(15)

using antioxidant pathways such as peroxiredoxin (Prx), thioredoxin/thioredoxin reductase (Trx/TrxR), or glutaredoxin (Grx). R1 and R2 can be H or small thiols such as cysteine (CysSH) or glutathione (GSH) [6].

### 2.4. Reactive Oxygen, Nitrogen, Sulfur Species (RONSS)

Although the reducing capacity of H_2_S could directly counteract the oxidizing capacity of · **·**NO and O_2_^−^ (Figure 5), the direct antioxidant action of H_2_S under physiological conditions does not seem particularly important. This is derived from the volatility and reactivity of H_2_S, making it a short-lived chemical species in cells, with HS^−^ and other RSS being more abundant. Therefore, the antioxidant action of H_2_S is indirect through the abovementioned interactions between RSS, RNS, and ROS [39,80].

Crosstalk has been shown to occur between RSS, RNS, and ROS; these interactions have been studied mainly in signaling molecules **·**NO, O_2_^−^, and H_2_S [60]. For example, H_2_O_2_ 10 mM induces the synthesis of **·**NO in leaf epidermal preparations of *Phaseolus aureus* [90], and during the induction of thermotolerance by applying H_2_O_2_ in corn seedlings, it was shown that H_2_O_2_ causes an increase in the synthesis of **·**NO, which, in turn, causes that of H_2_S [91]. With the stimulation of heat shock (45 °C for 30 min), *A. thaliana* plants sprayed with H_2_O_2_ (20–200 μM) increased **·**NO; **·**NO, in turn, stimulated the activity of catalase, ascorbate peroxidase, and glutathione reductase that eliminated excess H_2_O_2_, reducing the risk of oxidative damage [92]; **·**NO also favors the expression of the mitochondrial alternative oxidase under salt stress [93].

Similarly, the increase in endogenous H_2_S by the application of NaHS increased the activity and gene expression associated with catalase, superoxide dismutase, and peroxidase, reducing the oxidative damage induced by osmotic stress with 0.3 M mannitol [94], Cd toxicity [95], or Cr stress [96]. An analogous impact of the H_2_S donor GYY4137 by reducing **·**NO accumulation on stomata has been described [97]. Similarly, in tomato plants subjected to salinity, **·**NO functioned as an inducer of H_2_S synthesis, but not vice versa [98]. Otherwise, a study with barley seedlings subjected to salinity determined that the biostimulant impact of H_2_S depends on the endogenous synthesis of **·**NO [99]. However, the effects of H_2_S on ROS metabolism do not always occur through the promotion of antioxidant enzyme activity, as was demonstrated in peroxisomes, in which H_2_S is associated with catalase inhibition [100].

Subsequently, hormones such as auxin [101], melatonin [102], and salicylic acid [103] can function as downstream signaling in the biostimulation process and improve stress tolerance. It has also been found that the reverse is true and that applying gibberellic acid induces the endogenous synthesis of H_2_S, reducing oxidative damage by boron toxicity [104]. **·**NO has also been associated with plant responses to nanomaterials (NMs), either in the induction of tolerance to stress by NMs or in the plant response to stress caused by NMs [105].

RONSS crosstalk also occurs with other gasotransmitters. For example, it was reported that the favorable impact of H_2_ on cut flowers seems to be mediated by H_2_S, which decreases the expression of genes associated with senescence [106]. Similarly, the CO-dependent root architecture and the organogenesis of adventitious roots induced by CH_4_ depends on the induction of the synthesis of **·**NO and H_2_S [107,108]; the greater tolerance to stress caused by CH_4_ relies on the synthesis of **·**NO [109]. The crosstalk between RSS, RNS, and ROS and their subsequent impact on signaling molecules and growth regulators promote cell redoxtasis and could cause different molar ratios between the reactive species depending on the environmental factors and the cellular development context.

RONSS crosstalk also occurs with Se. Se is an element located in the same group as S, and like the latter, it also fulfills functions associated with redox homeostasis. Selenium is an essential element in mammals and macroalgae, with a broad spectrum of functions. One of the most studied functions is participating in antioxidant selenoproteins, which protect against oxidative stress and neutralize ROS and RNS. Selenoproteins contain selenocysteine and selenomethionine, and to date, the best-identified are those of the glutathione peroxidase (GPx), iodothyronine deiodinase, thioredoxin reductase, and selenophosphate synthetase families, which contribute to the maintenance of redoxtasis [110]. Furthermore, it has been established that the application of Se at low concentrations promotes stress tolerance, growth, and nutraceutical value [111] due to its impact on antioxidant enzymatic activity and the synthesis of redox-active metabolites.

It has been shown that the activity of glutathione peroxidase, ascorbate peroxidase, superoxide dismutase, dehydroascorbate reductase, and monodehydroascorbate reductase is increased [112,113]. These antioxidant enzymes directly impact ROS, and their effect on RNS and RSS is indirect, considering what has been exposed about the association between reactive species (Figure 5). Se has also been related to the increase in the activity of other non-catalytic proteins, such as thioredoxin (TrxR) and protein P [114]. The impact of Se on antioxidant metabolites is associated with sulfur metabolism since both elements share uptake and assimilation pathways; the effects on the concentration of GSH and GSSH have been described in *Allium* [115] and *Prunus domestica* [116]. In species that can reach high concentrations of S, such as broccoli, the accumulation of glucosinolates is increased [117].

Additionally, it has been shown that the presence of selenium increases the activity of phenylalanine ammonium lyase (PAL) and the accumulation of phenolic compounds, which due to their reducing capacity can modify the balance of RONSS [118]. The direct action of Se on redox homeostasis has also been proposed through the induction of antioxidant activity by spontaneous reduction of O_2_^−^ by GPx or by promoting the synthesis of ascorbic acid [119]. Another form of the direct action of Se is as a pro-oxidant, causing moderate oxidative stress with the formation of ROS that triggers the synthesis of enzymatic and non-enzymatic antioxidants [120].

Despite the close physiological and nutritional relationship between S and Se [121], the interaction between these elements in their impact on redoxtasis is poorly understood [122].

**Figure 6:**
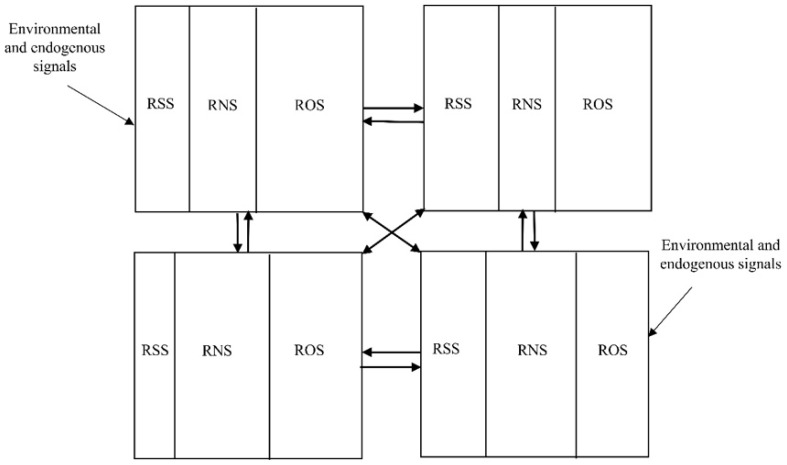
The dynamic balance in the relative amount of RSS, RNS, and ROS molecules is represented. In addition to the modifications of the RSS, RNS, and ROS species profiles, each change in the balance between the relative quantities (redoxtasis) would imply adjustments in the phenotype (transcriptome, proteome, metabolome, etc.) of the plant. The changes would be responses to external environmental signals, such as temperature and irradiance, and endogenous signals from the organism itself.

The adjustments in the molar balances between the different RSS, RNS, and ROS (Figure 6), as a result of various environmental stimuli and different physiological conditions, give rise, on the one hand, to the diversity of ratios between reactive species, metabolites, and enzymatic activities that define the cellular redoxtasis [123] and, on the other hand, to the presence of multiple proteomic [124] and metabolomic landscapes. The proteomic differences between individuals at different stages of development and/or in different environments or growth conditions are a consequence of the interaction of RSS, RNS, and ROS with cysteine residues or other amino acids such as tyrosine, which can be subjected to peroxidation, carbonylation, nitrosation, glutathionylation, persulfidation, sulfenylation, and sulfonylation [66,125].

## 3. RONSS as Biostimulants

From the point of view of biostimulation or priming with RONSS, the application of ROS, RNS, or RSS, or the use in pairs ROS–RNS, ROS–RSS, RNS–RSS constitutes a relevant and dynamic topic in plant science [50,57,60,61] (Table 4). In the same way, it is known that the mechanism of action of seed magnetopriming and some biostimulants, such as melatonin, salicylic acid, and silicon, includes the action of RONSS as signaling agents [102,103,126,127,128]. Although many examples are known where the application of RONSS induces favorable responses to stress, an increase in productivity or yield, or an improvement in nutritional composition in plants, there are still many gaps in knowledge about the molecular mechanisms involved in cellular responses [34,60,61]. The explanation of the above gaps lies in the great complexity of the interactions of the RONSS with the different cellular components [57,60].

Table 4 shows that coincidences occur in the proposed functions or impact on plants for the different reactive species. For example, the mitigation of electrolyte leakage and the decrease in lipid peroxidation can be achieved with the combination of ROS–RNS and RSS–RNS. Therefore, as confirmed by the studies cited in Table 4, the RONSS seems to function non-independently through crosstalk between the different signaling pathways [13,34,57,108], as depicted in Figure 5 and Figure 6. The mechanism that enables the RONSS to exert their effects in a coordinated way, as explained in the first section, is thought to have been the result of prebiotic evolution that had the goal of developing processes coordinated to obtain the maximum capacity for free energy processing and entropy production [8]. The biochemical descendants of that primordial processes are still active in cells. Through billions of years of biological evolution, natural selection adjusted and adapted them to permit the maximum capacity of the cells and multicellular organisms to process free energy and transform it into entropy [10].

The purpose of maximum entropy requires that organisms have a process for obtaining information that allows them to adjust to environmental changes, which is achieved by determining the energy condition through the evaluation of the redox status of the system [141], which can be equivalent to the variations in the molar ratios of the different reactive species (Figure 6). Information on redox status causes changes in gene expression and phenotype adjustments and proteomic and metabolomic responses that modulate the metabolism according to the organism’s needs in a particular environment. The RONSS are relevant messengers of the above metabolic adjustments [34].

The number of known chemical agents involved in cell signaling and biostimulation will likely grow as new information about other signaling molecules that work in coordination with RONSS is acquired. H_2_ and CO can be examples [108,142]. RONSS work in coordination with many other biomolecules, forming an intricate network of cellular information about energy status and responses to environmental stimuli [143,144]. The preceding points to the joint use of RONSS with biostimulants such as silicon, selenium, or iodine, plant and seaweed extracts, chitosan and other biopolymers, humic substances, and metabolites such as melatonin and salicylic acid [50,102,145,146,147,148,149].

As mentioned in Table 4, the application of RONSS for signaling and as a biostimulant has been evaluated in several plants with economic purposes, such as *Triticum aestivum*, *Solanum tuberosum*, *Citrus aurantium*, among others, which have shown promissory results. In this regard, early studies with exogenous application of sodium hydrosulphide (SHS) as a donor of H_2_S on *T. aestivum* seedlings under Cu stress showed an improvement in the activity of glutathione reductase, dehydroascorbate reductase, L-galactono-1,4-lactone dehydrogenase and gamma-glutamyleysteine synthetase. Moreover, the levels of ascorbic acid, glutathione, and total ascorbate increased, alleviating the damage produced by Cu [150]. Reduced damage of plasma membrane integrity in *T. aestivum* seeds exposed to Cu, promotion of amylase and esterase activities and lower levels of malondialdehyde, and H_2_O_2_ in germinating seeds treated with H_2_S donors have also been reported [151]. Tolerance against Cd stress in *T. aestivum* through the application of NO and H_2_S using sodium nitroprusside (SNP) and SHS as donors, respectively, showed an increase in dry matter, chlorophyll a and b, and Fv/Fm ratio between 39.1–47.8, 61.5–92.3, and 27.2–29.1, respectively, related to the control [152]. Under cobalt (Co) stress, *T. aestivum* exposed to Co concentrations of 150–300 µM and treated with NO and H_2_S donors showed an increase of glutathione (GSH), superoxide dismutase (SOD), peroxidase (POX), monodehydroascorbate reductase (MDHAR), APX, glutathione reductase (GR), dehydroascorbate reductase (DHAR), ascorbate (tAsA), and counteracted the negative effect caused by Co on growth, water relations, redox, and antioxidant capacity in chloroplasts [51]. The addition of SNP (100 µM) as a donor of NO in *T. aestivum* has also been demonstrated to counteract the negatives effects of 400 µM Fe, enhanced seed germination, decreasing Fe accumulation, and proline and malondialdehyde (MDA) content [153]. Under water deficit conditions, RONSS application has also demonstrated that *T. aestivum* seeds can mitigate the damage produced by water scarcity. The seeds soaked with SNP (0.1 mM) or H_2_O_2_ (1 mM) or a combination of both improved Ψw, Ψs, Ψp, photosynthetic pigment content, osmolytes accumulation (GB and Pro), TSP, and the antioxidative defense mechanism. Moreover, it also reduced MDA accumulation [154].

Other species with commercial importance, such as *Citrus aurantium* or *Solanum lycopersicum* have also been evaluated. In this regard, adverse effects caused by salinity stress (120 mM NaCl) on *S. lycopersicum* (47% of decrease in dry leaf mass and root length) were alleviated by exogenous application of SNP (100 µM) enhanced the leaf dry mass (30%) and root length (23%) compared with the non-treated plants [155]. NO has been associated with root development in *S. lycopersicum* growing under elevated CO_2_ concentration, especially in lateral roots, and increasing nitric oxide synthase activity [156]. SNP applied as NO donor at 100 µM in *S. lycopersicum* showed a good capacity to immobilize As in the root but also its translocation in the shoots by upregulation of γ-glutamylcysteine synthetase (GSH1), glutathione synthetase (GSH2), phytochelatin synthase (PCS), metallothionein (MT), and ABC transporter (ABC1). Interestingly, the authors reported that the plants subjected to As stress (10 mg/L) and treated with SNP were able to restore the growth retardation through modulating the chlorophyll and proline metabolism, with an increase of stomatal conductance and NO accumulation [157]. Studies carried out with *Citrus aurantium* have also demonstrated how nitrosative and oxidative signals play an important role in regulating cellular adjustments to environmental conditions. In this regard, plants subjected to salinity stress (150 mM NaCl) and pre-treated with H_2_O_2_ (10 mM for 8 h) and SNP (100 µM for 48 h) showed a strong reduction of phenotypical and physiological effects, as well as a higher net photosynthetic rate compared with the non-treated plants that showed clear foliar injury (necrosis) and low net photosynthetic rates [140]. Moreover, these same authors reported that proteomics analysis reveals quantitative variations in 85 leaf proteins in plants subjected to salinity. Many of these were not present in H_2_O_2_ or SNP pre-treated plants. Histochemical and fluorescent probes in *C. aurantium* plants pre-treated with H_2_O_2_ and SNP showed ROS movement by vascular tissues over long distances and NO signaling pathways [125].

In other species, such as *Solanum tuberosum*, the use of NO donors (SNP, S-nitroso-N-acetylpenicillamine or a mixture of ascorbic acid and NaNO_2_) demonstrated that NO could protect plants from methylviologen damage produced by herbicides [158], but could also stimulate phytoalexin accumulation, which can be used as a mechanism of induction of defense against pathogens in plants [159] or to participate in the wound–healing response of potato leaves by the induction of cell wall glucan callose production [160]. On the other hand, since H_2_O_2_ is relatively stable compared to other ROS molecules such as NO, a recent study demonstrated that foliar spraying of H_2_O_2_ at 1% consecutively (7 days) on *S. tuberosum* caused an increase in the photosynthetic apparatus and antioxidant capacity [161].

Strawberries are a highly demanded fruit consumed globally, known for their biological properties such as antioxidant, antimicrobial, or anti-inflammatory capacity [162]. In early studies developed with *Fragaria* × *ananassa* it was demonstrated that fumigation for 5 h with NO at 200 µL/L NO atmospheres and maintained at 18 °C in air delayed the onset of ethylene production and reduced the respiration, maintaining the fruit’s quality and prolonging its shelf life [163]. Similar results were obtained fumigating *F.* × *ananassa* with NO (between 1.0 to 4000 µL L^−1^) immediately after harvest and held at 5 °C and 20 °C in air containing 0.1 µL L^−1^ [164]. At both temperatures, the postharvest life of *F*. × *ananassa* was extended, but the optimal NO concentration was 5–10 µL L^−1^, causing > 50% extension in shelf life. The application of sodium hydrosulfide (NaHS) as a donor of H_2_S on *F*. × *ananassa* under iron deficiency has also been evaluated [165]. Leaf interveinal chlorosis caused by iron deficiency was overcome by foliar application of NaHS. Moreover, applying H_2_S donors enhanced chlorophyll contents and iron accumulation in young leaves. However, the H_2_S enhanced not only iron deficiency but also the assimilation of other micronutrients such as Zn, Ca, and Mg [166]. Iron deficiency in *F*. × *ananassa* concomitant with salinity stress (50 mM NaCl) has also been overcome by the exogenous application of NO through SNP as a donor. SNP applied at 0.1 mM showed that plants under iron deficiency and salinity reduced the exacerbated electrolyte leakage, malondialdehyde levels, and H_2_O_2_ levels caused by the stress [165]. In recent work, [167] determined that applying SNP as NO donor at 100 µM alleviated heat injury in *F.* × *ananassa* plants. NO controlled the overaccumulation of H_2_O_2_, reduced lipid peroxidation, and improved the relative water content and a higher expression of heat shock transcription factor genes involved in thermotolerance. According to the information shown above, NO or H_2_S are gaseous signaling molecules with an important role in response to diverse biotic and abiotic stresses in plants, regulating normal plant growth and development. This evidence suggests that RONSS are a potential tool for use in the biostimulation of crops.

The RONSS studies for their potential as signaling molecules or biostimulants have also been evaluated in medicinal plants. Although they have been less studied, medicinal plants have also been used as a model in some assays. In this regard, *Catharanthus roseus*, an endemic medicinal plant from Madagascar, was used as a model to evaluate its tolerance to metal stress in the presence of NO [168]. The plants were exposed to 30 mg kg^−1^ of Cu (CuCl_2_·2H_2_O) alone or mixed with SNP as a donor of NO in concentrations of 0–400 µM. The results showed that the damages produced by Cu in *C. roseus* (Cu^+2^ accumulation, decrease in NO production, disruption in mineral equilibrium, and high ROS production) were alleviated by SNP presence and in a more significant proportion by 50 µM of SNP. Moreover, the treatment with SNP and Cu + SNP significantly prevented or restored the Cu-induced depression of iron in the root. In addition, interestingly, the authors found that the application of SNP caused an increase in leaf vincristine and vinblastine, two potential anticancer compounds [169], which have been previously reported in *C. roseus* [170].

*Artemisia annua* is an important vegetal source against malaria [171]. Adverse effects caused by Cu^+2^ (20 to 40 mg kg^−1^) on *A. annua* can be alleviated by exogenous application of H_2_S (200 µM), restoring physiological and biochemical parameters, reducing lipid peroxidation and enhancing the antioxidant activity of plants [172]. Additionally, H_2_S application increased the photosynthetic efficiency and trichome density and the production of artemisinin content [171], a well-known compound used against malaria, but also with anti-inflammatory, antioxidant, and antimicrobial effects [173].

H_2_S has also been effectively used in *Carthamus tinctorius*, an Asteraceae with essential medicinal properties and a source of food-grade color in the food industry [174]. The exogenous application of H_2_S (1 mM) on *C. tinctorius* plants subjected to drought demonstrated that the harmful effects caused by the water scarcity were countered, increasing the accumulation of secondary metabolites and antioxidant capacity [175]. Exogenous application of SNP as a NO donor on *Gingko biloba* at different concentrations (50, 100, 250, and 500 μM) demonstrated that the high concentrations (500 μM) favored the increase of phenolic compounds, glycosides, tannins, and saponins. Moreover, a significant increase in an oxidative burst of O_2_^−^ was also detected, enhanced phenylalanine ammonia-lyase (PAL) activities and antioxidant defense enzymes such as superoxide dismutase and ascorbate peroxidase [176]. Similar results were obtained in *G. biloba* by applying 250 μM L^−1^ of SNP under drought stress. The authors reported that after the treatment with SNP, remarkably soluble sugar, proline, flavonoid, and ginkgolide content was obtained in *G. biloba* leaves, as well as increased PAL activity, demonstrating the capacity of NO to alleviate the adverse effects caused by drought stress [177].

Another medicinal plant is *Silybum marianum*, which is used to treat liver and biliary disorders. *S. marianum* contains silymarin, a mixture of flavonoid complexes with a protective component against drugs, including chemotherapy [178]. Field assays with two genotypes of *S. marianum* demonstrated that applying the SNP (100 µM) as a NO donor compensates for 40% of the adverse effects caused for drought stress, and all yield components responded significantly to treatment with SNP [179]. Applying 100 µM SNP also decreased malondialdehyde content and H_2_O_2_ in *S. marianum* plants submitted to water deficit and prevented a silymarin yield reduction but increased taxifolin production, silychristin, silybin, and isosilybin B [180], compounds that have been associated with the treatment of diseases due to pharmacological properties as hepatoprotective drugs [181,182]. Under drought stress applying 100 µM SNP on *S. marianum*, the leaf photosynthesis rate increased between 80 and 100% compared with the non-treated plants [179].

Ginsenosides are compounds associated with rhizomes and roots of *Panax ginseng*. It has a therapeutic potential as an adjuvant in treating diabetes mellitus [183]. In this regard, using SNP as a NO donor, together with methyl jasmonate and applied in adventitious roots of *P. ginseng*, has shown that a high concentration of ginsenoside was obtained with 200 µM SNP. Additionally, the application of 200 µM SNP and 100 µM methyl jasmonate caused a high induction of ginsenoside biosynthesis-related genes and detected a high sensitivity of the superoxide dismutase 1 gene [184]. In another interesting work, [185] reported stimulatory responses in *Origanum majorana* German type under drought stress and treated with SNP at 30 and 60 µM. Its application enhanced the growth and yield of essential oil, improved water use efficiency, and caused an upregulation in the antioxidant system. Interestingly, the use of SNP also caused a significant increase in the production of phytopharmaceuticals (total soluble phenol, anthocyanin, flavonoids, and ascorbic acid) in the herbal extract. As mentioned above, most studies have been performed under drought conditions. However, using NO has also caused stimulatory effects in medicinal plants under salt stress. In this regard, [186] developed a study to evaluate the use of NO and spermidine, a known polyamine protector of plants [187], as pretreatment of *Matricaria recuita* plants. The results showed increased growth parameters, significant malondialdehyde and H_2_O_2_ content reduction, and increased ascorbate peroxidase activity.

Finally, it is essential to mention that medicinal plant extract’s biological efficacy in preventing oxidative damage is well documented [188,189,190]. However, their capacity as free radical scavenging or as biostimulant agents favoring the RONSS formation or the increase of antioxidant enzymes has been focused mainly on treating human inflammation or wounds [189,191]. On the other hand, we cannot ignore that plant-derived extracts can act as biostimulants in sustainable agriculture. The systematic application of plant-based products has been shown to promote plant growth and improve damage caused by environmental stresses, which has been associated with the presence of polysaccharides, polyphenols, vitamins, phytohormones, etc. [192,193]. In this regard, recent excellent reviews have focused on the role of moringa leaf as a plant biostimulant to improve the quality of agricultural products [194,195]. Hydrolysate-based biostimulants from *Medicago sativa* containing triacontanol and indole-3-acetic acid have been reported to stimulate the growth of *Zea mays* under salinity stress [196]. Since this review was focused only on RONSS species and their use as signaling molecules or biostimulant agents, this aspect will not be addressed in detail, but for more information, see [197] and [193].

NO is a labile molecule and challenging to apply in an exogenous way due to its gaseous nature and short in vivo half-life (between 1 and 5 s). NO has been successfully applied in maize to alleviate the damage produced by saline stress [198]. The authors used chitosan nanoparticles containing the NO donor S-nitroso-mercaptosuccinic acid as a carrier. As a result, a sustained NO release was reported, and amelioration of the harmful effects of salinity on the photosystem II activity, chlorophyll content, and growth of maize plants was observed [198]. In this same way, NO release from chitosan nanoparticles containing S-nitrosoglutathione (GSNO) as an NO donor was demonstrated to attenuate the effects of water deficit on sugarcane plants [199]. Furthermore, encapsulating GSNO into chitosan nanoparticles was shown to cause higher photosynthetic rates under water deficit, and increased the root/shoot ratio.

From a practical point of view, it can be thought that considering the great availability in the atmosphere and the ease of absorption of O_2_ by plants through stomata and lenticels, the presence of ROS in plant cells will always be ensured at the necessary quantities. The above considers the many mechanisms and environmental factors associated with ROS synthesis (Figure 3). However, despite the potential abundance of ROS in plant cells, different studies show that priming with ROS yields favorable results in different plant species [53,57,136].

On the other hand, unlike ROS, RNS and RSS are not obtained from a resource as abundant as O_2_. Instead, both RNS and RSS are synthesized from plant nutrients whose greater volume is assimilated by the root in the form of NO_3_^−^, NH_4_^+^, SO_4_^2+^, and amino acids. In addition to being much smaller than those of O_2_ in volume, these nutrients require a previous absorption, transport, and assimilation process to produce the necessary RNS and RSS. The above implies the possibility that to obtain biostimulation with RONSS, only the exogenous application of RNS and RSS or the precursors of **·**NO and H_2_S is necessary. It is even considered that the proper use of fertilizers with N and S can provide the amounts of RNS and RSS essential to achieving improvement in signaling and stress tolerance in plants or obtaining a more significant impact with the use of biostimulants, such as the use of elemental sulfur (S^0^) or organic fertilizers with S^2−^ [79,200]. In the case of S, a regular supply of fertilizers is necessary, since repeated crop extractions and continuous land tillage that oxidizes soil organic matter cause a decrease in soil S stores [201].

A scheme similar to the one previously mentioned was presented in the study by [202], who used 100 μM **·**NO (as donor sodium nitroprusside) in combination with split applications of N and S fertilizers (50 + 50 mg kg^−1^, two times) in plants of *Brassica juncea*. The results showed that the combination **·**NO+N+S significantly promoted photosynthesis, stomatal performance, and growth in the absence of salt stress and meaningfully alleviated the impact of salt stress through increased proline, N- and S-use efficiency, and antioxidant system. Presumably, using **·**NO in combination with the N and S fertilizer sources allowed an adequate balance of RNS and RSS.

## 4. Conclusions

RONSS exert their functions by interacting with many biomolecules forming a complex cellular information network that indicates the energy status of the system and regulates responses to environmental stimuli.

The use of RONSS as biostimulants in plants is feasible and practical, using techniques such as adequate fertilization with N and S and the use of tolerance-inducing biostimulants such as silicon, organic acids, or chitosan or with the application of precursors of RNS and RSS combined with direct application of ROS, e.g., H_2_O_2_. In this sense, applying exogenous NO incorporated in chitosan nanoparticles has proven to be a feasible alternative for alleviating the adverse effects in plants caused by abiotic stress. However, few works have been developed, and more in-depth studies are necessary.

The use of RONSS as biostimulants significantly modifies the phenotype and metabolic activity of plants since RONSS has impacts on and interactions with the main metabolic pathways such as photosynthesis, respiration, the flow of water, and nutrients, as well as with other signaling molecules, such as hormones.

Knowledge about the integration of interactive networks between ROS, RNS, and RSS and between RONSS and other signaling biomolecules is still incomplete. The enormous complexity of the processes, the mutual interactions between the system’s components, and the emergent properties that result from the system´s components´ interactions do not allow a simple approach to the functional scheme in which the RONSS are incorporated.

## Figures and Tables

**Table 1:** Representative oxygen compounds and their oxidation state. ROS are in bold letters.

Oxidation State	Representative Compound and Formula
+2	OF_2_
+1	O_2_F_2_
0	O_2_
−1/2	All superoxides, O_2_^−^, **O_2_^−^**, **HO_2_**
−1	All the peroxides, **H_2_O_2_**, **HO_2_^−^**, **HO**
−2	All the oxides, H_2_O, CO_2_

**Table 2:** Representative nitrogen compounds and their oxidation state. RNS are in bold letters.

Oxidation State	Representative Compound and Formula
+5	HNO_3_, NO_3_^−^, **ONOO^−^ (peroxynitrite)**
+4	**·NO_2_ (nitrogen dioxide)**, N_2_O_4_
+3	HNO_2_, NO_2_^−^, **S-nitrosothiols (RS-N=O)**, **NO^+^**
+2	**·NO (nitric oxide)**
+1	N_2_O (nitrous oxide), **NO^−^**
0	N_2_
−1	NH_2_OH (hydroxylamine)
−2	N_2_H_4_ (hydrazine)
−3	NH_3_, NH_4_^+^

**Table 3:** Representative sulfur compounds and their oxidation state. RSS are in bold letters. Modified from [79].

Oxidation State	Representative Compound and Formula
+6	Sulfate, SO_4_^2−^
+6 and −2	Thiosulfate, S_2_O_3_^2−^
+5 and −2	Polythionates (-O_3_S-Sn-SO_3_-)^2−^ Dithionate, S_2_O_6_^2−^ Trithionate, S_3_O_6_^2−^ Tetrathionate, S_4_O_6_^2−^
+4	Sulfur dioxide, SO_2_ Sulfite, SO_3_^2−^ Disulfite, S_2_O_5_^2−^ Sulfonic acid (RSO_3_H) from ROS-mediated protein sulfonylation. Sulfone, OS(S) the oxidation product of sulfoxides.
+3	Dithionite, S_2_O_4_^2−^ **Disulfide-S-dioxide (thiosulfonate) RS(O_2_)SR**
+2	Carbonyl Sulfide (COS), OCS. **Sulfinic acid (RSO_2_H)** from ROS-mediated protein sulfinylation.
+1	**Disulfide-S-monoxide (thiosulfinate) RS(O)SR**
0	S^0^ (sulfane sulfur), elemental sulfur, mainly S_8_ (cycloocta-S). Sulfoxide (R-S(-O)-R such as the dimethyl sulfoxide (DMSO). Oxidized derivatives of sulfide and **sulfenic acid (RSOH)** from ROS-mediated protein sulfenylation. Near the six electrons, valence S^0^ never exists by itself. Sulfane sulfur (S^0^, S-S, or S_2_) is labile. There are a variety of compounds such as S_8_, thiosulfate, polysulfanes, and polysulfides, that contain S^0^
−1	**Disulfide (RSSR)** is a persulfide ^−^S-S^−^ found in the linkages between two cysteine residues in proteins. RSSH denotes persulfides (also called hydrosulfides or hydropersulfides) obtained by the action of H_2_S on cysteine residues **(R-SH)**. Thioethers and thiols can be oxidized to disulfides. Persulfides such as CysSSH, GSSH, and protein-SSH act as signaling compounds in organisms. Major products of the decomposition of persulfides are polysulfanes **Disulfide-S-monoxide (thiosulfinate) RS(O)SR** **Disulfide-S-dioxide (thiosulfonate) RS(O_2_)SR** **Thiyl-radical HS· or RS·**
−2	**Sulfide, S_2_^−^ and organic polysulfides, S_2_^2−^, S_3_^2−^, S_5_^2−^** **Disulfides (R-S-S-R)** Carbon disulfide (CS_2_) FeS_2_ NaHS and Na_2_S are sources of S_2_^−^ and of its conjugated acids SH^−^ and **H_2_S**. Organic and inorganic polysulfides (with Sn > 2) contain S^0^ atoms, which allows a diversity of oxidation states.
−2	**Hydrogen sulfide (H_2_S), disulfane or hydrogen persulfide (H_2_S_2_), H_2_S_3_, other inorganic polysulfides (H_2_S_x_) x ≥ 1**, and polysulfanes (RSS_n_H, RSS_n_SR, *n* > 2). Polysulfanes contain S^0^ atoms, which allows a diversity of oxidation states.
−2	Thioethers (C-S-C) such as dimethyl sulfide (DMS), CH_3_-S-CH_3_ and dimethyl disulfide (DMDS), CH_3_-S-S-CH_3_.
−2	**Thiols (R-SH)** such as glutathione (GSH) and methyl mercaptan, CH_3_-SH. Thiols are derived from the sulfhydryl group -SH of cysteine that enables multiple oxidation states (−2 to +6). Thiolates are anionic derivatives of thiols in which a metal or other cation replaces H.

**Table 4:** Some examples of studies where the favorable impact of applying at least two of the reactive species: ROS, RNS, and RSS or their precursors in plants has been demonstrated.

Impact on the Plant	Reactive Species	Plant Species	Reference
Decreased absorption and/or toxicity of heavy metals	H_2_S, **·**NO H_2_S, **·**NO H_2_S, **·**NO H_2_S, **·**NO	*Medicago sativa* *Sesamum indicum* *Triticum aestivum* *Triticum aestivum*	[129] [130] [131] [51]
Increase in the concentration of essential elements	H_2_S, **·**NO H_2_S, **·**NO	*Triticum aestivum* *Sesamum indicum*	[131] [130]
Increase in Relative Growth Rate (RGR) and/or biomass	H_2_S, **·**NO H_2_S, **·**NO H_2_S, **·**NO H_2_S, **·**NO H_2_S, **·**NO H_2_S, **·**NO H_2_O_2_, **·**NO H_2_O_2_, **·**NO H_2_O_2_, **·**NO	*Cynodon dactylon* *Medicago sativa* *Sesamum indicum* *Solanum lycopersicum* *Triticum aestivum* *Triticum aestivum* *Ocimum basilicum* *Oriza sativa* *Triticum aestivum*	[129] [132] [130] [133] [51] [131] [134] [135] [136]
Improved crop yield and/or quality	H_2_O_2_, **·**NO	*Ocimum basilicum*	[134]
Increase in Relative Water Content (RWC)	H_2_S, **·**NO H_2_O_2_, **·**NO	*Triticum aestivum* *Fragaria × ananassa*	[51] [137]
Increment in stomatal conductance (gs)	H_2_S, **·**NO H_2_S, **·**NO	*Medicago sativa* *Triticum aestivum*	[50] [51]
Increase in the quantum efficiency of PSII (Fv/Fm)	H_2_S, **·**NO H_2_S, **·**NO H_2_O_2_, **·**NO H_2_O_2_, **·**NO	*Medicago sativa* *Triticum aestivum* *Citrus aurantium* *Fragaria × ananassa*	[50] [51] [125] [137]
Increase in CO_2_ assimilation (A)	H_2_O_2_, **·**NO	*Citrus aurantium*	[125]
Increment in the concentration of photosynthetic pigments	H_2_S, **·**NO H_2_S, **·**NO H_2_O_2_, **·**NO H_2_O_2_, **·**NO H_2_O_2_, **·**NO	*Sesamum indicum* *Triticum aestivum* *Citrus aurantium* *Fragaria × ananassa* *Ocimum basilicum*	[130] [131] [125] [137] [134]
Increased activity of antioxidant enzymes (e.g., SOD and CAT) and the ascorbate–glutathione (AsA–GSH) cycle	H_2_S, **·**NO H_2_S, **·**NO H_2_S, **·**NO H_2_S, **·**NO H_2_S, **·**NO H_2_S, **·**NO H_2_S, **·**NO H_2_O_2_, **·**NO	*Cynodon dactylon* *Medicago sativa* *Medicago sativa* *Medicago sativa* *Solanum lycopersicum* *Triticum aestivum* *Triticum aestivum* *Ocimum basilicum*	[132] [138] [129] [50] [133] [131] [51] [134]
Proteome reprogramming through reversible or irreversible posttranslational modifications (PTM) and changes in gene expression	H_2_S, **·**NO H_2_S, **·**NO H_2_O_2_, **·**NO	*Citrus aurantium* *Citrus aurantium* *Citrus aurantium*	[139] [140] [124]
Mitigation of the relative electrolyte leakage under stress	H_2_S, **·**NO H_2_O_2_, **·**NO H_2_O_2_, **·**NO H_2_O_2_, **·**NO	*Cynodon dactylon* *Citrus aurantium* *Citrus aurantium* *Fragaria × ananassa*	[132] [124] [125] [137]
Mitigation of lipid peroxidation under stress	H_2_S, **·**NO H_2_S, **·**NO H_2_S, **·**NO H_2_S, **·**NO H_2_S, **·**NO H_2_O_2_, **·**NO	*Cynodon dactylon* *Medicago sativa* *Medicago sativa* *Solanum lycopersicum* *Triticum aestivum* *Fragaria × ananassa*	[132] [50] [129] [133] [131] [137]
Increased accumulation of proline and other osmolytes	H_2_S, **·**NO H_2_O_2_, **·**NO	*Medicago sativa* *Triticum aestivum*	[50] [136]

## Data Availability

Data sharing is not applicable to this article, as no datasets were generated or analyzed during the current study.
